# Validation of the preoperative controlling nutritional status score as an independent predictor in a large Chinese cohort of patients with upper tract urothelial carcinoma

**DOI:** 10.1002/cam4.1902

**Published:** 2018-11-28

**Authors:** Hang Xu, Ping Tan, Xi Jin, Jianzhong Ai, Tianhai Lin, Haoran Lei, Lu Yang, Qiang Wei

**Affiliations:** ^1^ Department of Urology, Institute of Urology West China Hospital, Sichuan University Chengdu China

**Keywords:** biomarker, controlling nutritional status, prognosis, radical nephroureterectomy, upper tract urothelial carcinoma

## Abstract

**Background:**

Pretreatment controlling nutritional status (CONUT) score is a novel index which was used to predict outcomes in cancer patients. We aim to explore the prognostic significance of CONUT score in patients with upper tract urothelial carcinoma (UTUC) after radical nephroureterectomy (RNU).

**Patients and methods:**

A total of 662 UTUC patients between 2004 and 2016 were retrospectively analyzed. Patients were categorized into three groups based on CONUT score (Normal: 0‐1; Light: 2‐4; Moderate/severe: 5‐12). Associations of CONUT score with oncological outcomes were analyzed using Logistic and Cox regression analysis. Harrell concordance index was used to assess the predictive accuracy of the multivariate models. Subgroup analyses were conducted according to tumor grade and stage.

**Results:**

The median follow‐up duration was 41 months. Multivariate Logistic analysis showed that high CONUT score was independently associated with high‐grade disease, high pT stage, lymphovascular invasion, sessile carcinoma, variant histology, and positive surgical margins (each *P < *0.05). Multivariate analysis demonstrated that CONUT score 5‐12 was an independent factor for worse cancer‐specific survival (CSS, hazard ratio [HR]:2.39, 95% confidence interval [CI] 1.55‐3.68, *P* < 0.0001), disease recurrence‐free‐survival (RFS, HR: 1.80, 95% CI 1.24‐2.60, *P* = 0.002), and overall survival (OS, HR: 2.26, 95% CI 1.53‐3.34, *P* < 0.0001). The estimated c‐index of the multivariate models for CSS, RFS, and OS increased from 0.755, 0.715 and 0.745 to 0.772, 0.723, and 0.756 when CONUT score supplemented. Subgroup analyses showed that especially in patients with high‐grade carcinoma and advanced stage (≥pT3), higher CONUT score predicts decreased CSS, RFS, and OS (all *P* < 0.05).

**Conclusion:**

Preoperative CONUT score is a negative independent prognostic indicator for both pathologic and survival outcomes in UTUC, especially in those with high‐grade carcinoma and advanced stage. Adding this parameter into our clinical prediction model is appropriate so as to improve its predictive accuracy.

## 1 INTRODUCTION

Upper tract urothelial carcinoma (UTUC) consists of ureteral carcinoma and renal pelvic carcinoma and occurs at a low frequency, accounting for roughly 5%‐10% of urothelial carcinomas in western population.^1^ Patients diagnosed with UTUC usually have worse prognosis in comparison of other genitourinary cancer (bladder cancer, prostate cancer, and renal carcinoma) despite undergoing radical nephroureterectomy (RNU) with bladder cuff resection, the standard procedure currently, with the 5‐year survival rate <50% in muscle‐invasive UTUC.^1^ Therefore, identifying prognostic factors which might allow effective interventions before or after surgery is urgent so as to prolong patients’ life expectancy.

In addition to the established pathologic prognostic indicators such as tumor stage and grade, recent interests have been switched to focus on exploring preoperative novel prognostic biomarkers in UTUC. Given the potential role of systematic inflammation and nutrition status in tumor progression and their associations with oncological outcomes,^2^, ^3^ several biomarkers that reflect the above two status have been explored in UTUC including neutrophil to lymphocyte ratio,^4^ albumin to globulin ratio,^5^ nutritional index,^6^ and controlling nutritional status (CONUT) score.^7^ Of them, CONUT score, a novel biomarker which is calculated from serum albumin, total lymphocyte counts, and total cholesterol concentrations, has shown its independent prognostic significance in various solid tumor types such as hepatocellular carcinoma,^8^ gastric cancer,^9^, ^10^ and colorectal cancer.^11^ It reflects individuals’ nutrition status, immunological function, protein and lipids metabolism. Ishihara et al^7^ first described the prognostic significance of pretreatment CONUT score in 107 UTUC sets of Japanese origin. They found that CONUT score was an independent predictor of cancer‐specific survival (CSS) and overall survival (OS) in UTUC patients after RNU.

Thus, we sought to further explore and validate the prognostic value of CONUT score in a large Chinese cohort of patients with UTUC. We also tend to assess whether it would improve the predictive accuracy in multivariate models for UTUC prognosis.

## 2 MATERIALS AND METHODS

### 2.1 Study population

This retrospective study received the approval from the Ethical Committee of West China Hospital. A total of 801 patients diagnosed with UTUC who had undergone RNU at our center from January 2004 to December 2016 were retrospectively reviewed. We excluded 32 patients without available clinicopathological and laboratory information, 18 patients with autoimmune disease/hepatic disease, 11 patients with non‐urothelial carcinoma, and 58 patients lost at the beginning of follow‐up. No patients had received neo‐adjuvant chemotherapies. The remaining 662 cases were included in our study for further analysis.

Open and laparoscopic RNU combined with open bladder cuff excision were performed at our department. Lymph node dissection was performed when suspected enlarged lymph nodes (identified by the preoperative radiology or intraoperative inspection).

Each patient’s information was retrieved from their medical records. Pathologic specimens were reevaluated by two experienced pathologists. The World Health Organization (WHO)/International Society of Urologic Pathology classification of 2004 was used to determine tumor grade, and the 2002 Union for International Cancer Control (UICC) TNM classification system was applied to confirm tumor stage. Pathological features including tumor architecture (sessile or papillary), lymphovascular invasion (LVI), positive surgical margins (PSM), and concomitant variant histology (CVH, urothelial carcinomas accompanying abnormal histological differentiation: eg, squamous cell) were simultaneously retrieved from the corresponding pathological reports. Tumor size was confirmed through radiology (Computed tomography/magnetic resonance imaging) or surgical specimens.

### 2.2 CONUT score definitions

The laboratory information was obtained within 2 weeks before surgery. The following three indicators: serum albumin, total lymphocyte counts, and total cholesterol concentrations were used to calculate CONUT score (based on published reports,^8^, ^11^ see Table1). Patients were divided into three groups according to their CONUT score: normal group: score 0‐1; light group: score 2‐4; and moderate/severe group: score 5‐12.

**Table 1:** CONUT scoring system according to the combination of serum albumin, total lymphocyte count and total cholesterol

Parameter	Normal	Light	Moderate	Severe
Serum albumin, g/L	≥35.0	30.0‐34.9	25.0‐29.9	<25.0
Score	0	2	4	6
Total lymphocyte count, /mm^3^	≥1600	1200‐1599	800‐1199	<800
Score	0	1	2	3
Total cholesterol, mg/dL	≥180	140‐179	100‐139	<100
Score	0	1	2	3
Total score (CONUT score)	0‐1	2‐4	5‐8	9‐12

CONUT, controlling nutritional status.

### 2.3 Follow‐up

Physical examination, urinary tests, and blood laboratory tests were routinely performed. Cystoscopy was done every 3 months for the first year after RNU, every 6 months for the next 2 years, and then once a year thereafter. Computed tomography or magnetic resonance imaging was performed every year or when suspected disease recurrence. Postoperative treatment regimens mainly included adjuvant chemotherapy (intravesical chemotherapy and/or systematic chemotherapy) and adjuvant radiotherapy, which were administrated according to tumor stage, nodal involvement, doctor’s selection, patients’ conditions, and desire. Intravesical chemotherapy drugs were mainly mitomycin C and pirarubicin. Systematic chemotherapy drugs were platinum‐based regimens. And there was one patient who had received immunotherapy. Disease recurrence was defined as recurrence from the operating site, lymph nodes, and visceral metastasis (to clarify: intravesical recurrence was not classified into disease recurrence). CSS was defined as the time in months from the date of RNU to cancer‐related death. Disease recurrence‐free survival (RFS) was defined as the time in months from RNU to disease recurrence. OS was defined as the time in months from RNU to death from all cause.

### 2.4 Statistical analysis

The Mann‐Whitney *U* test and Chi‐squared test were used to evaluate the continuous variables and dichotomous variables, respectively. Multivariate Logistic regression analysis was used to evaluate associations of CONUT score with adverse pathological outcomes after adjusting preoperative confounders. Probabilities of CSS, RFS, and OS were estimated by the Kaplan‐Meier method (Log‐rank tests were selected) and subgroup analyses were conducted according to tumor grade and tumor stage.

Cox proportional hazard regression models were used to identify the risk factors for CSS, RFS, and OS. Variables with a *P* value <0.05 in our univariate analysis were included in the multivariate model by using backward stepwise procedure (*P* < 0.2 for entry; *P* < 0.15 to remain). The c‐index was calculated to assess the model’s predictive accuracy. A two side *P* value <0.05 was considered statistically significant. Statistical analyses were carried out using R software (version 3.4.4) and IBM SPSS Statistics version 22.0 (IBM Corp., Armonk, NY, USA).

## 3 RESULTS

### 3.1 Baseline characteristics

Table 2 showed patients’ characteristics in this study. The 662 cases included 376 (56.8%) male and 286 (43.2%) female patients, and median patients’ age was 67 years (interquartile range [IQR]: 59‐74 yr). There were 484 (73.3%) patients with the body mass index (BMI) <25 kg/m^2^ and 176 (26.3%) with BMI ≥25 kg/m^2^. Patients were categorized into the following three groups: Normal (n = 270, 40.8%), Light (n = 302, 45.6%), Moderate/severe (n = 90, 13.6%). There were no differences between groups with regard to age, gender, BMI, tumor side, hydronephrosis, multifocality, surgical approach, lymph node status, tumor size, and adjuvant radiotherapy (each *P* > 0.05). In contrast, we can observe the differences between groups considering tumor location, tumor grade, pT stage, LVI, PSM, tumor architecture, CVH, and adjuvant chemotherapy (Table 2).

**Table 2:** Baseline characteristics of the patients included this study

Variables	Total	Normal (0‐1)	Light (2‐4)	Moderate/severe (5‐12)	*P*
	662	270 (40.8)	302 (45.6)	90 (13.6)	
Age (y)					
<65	254 (38.4)	104 (38.5)	122 (40.4)	28 (31.1)	0.282
≥65	408 (61.6)	166 (61.5)	180 (59.6)	62 (68.9)	
Gender					
Male	376 (56.8)	148 (54.8)	176 (58.3)	52 (57.8)	0.692
Female	286 (43.2)	122 (45.2)	126 (41.7)	38 (42.2)	
BMI					
<25	484 (73.3)	193 (71.5)	220 (72.8)	71 (78.9)	0.388
≥25	176 (26.7)	77 (28.5)	80 (27.2)	19 (21.1)	
Tumor side					
Left	341 (51.5)	148 (54.8)	148 (49.0)	45 (50.0)	0.364
Right	321 (48.5)	122 (45.2)	154 (51.0)	45 (50.0)	
Hydronephrosis					
No	247 (37.3)	91 (33.7)	124 (41.1)	32 (35.6)	0.180
Yes	415 (62.7)	179 (66.3)	178 (58.9)	58 (64.4)	
Tumor location					
Pelvicalyceal	349 (52.7)	136 (50.4)	160 (53.0)	53 (58.9)	0.011
Ureteric	193 (29.2)	96 (35.6)	80 (26.5)	17 (18.9)	
Both	120 (18.1)	38 (14.1)	62 (20.5)	20 (22.2)	
Multifocality					
No	550 (83.1)	225 (83.3)	244 (80.8)	81 (90.0)	0.122
Yes	112 (16.9)	45 (16.7)	58 (19.2)	9 (10.0)	
Surgical approach					
Open RNU	430 (65.0)	175 (64.8)	194 (64.2)	61 (67.8)	0.825
Laparoscopic RNU	232 (35.0)	95 (35.2)	108 (35.8)	29 (32.2)	
Tumor grade					
Low	169 (25.5)	91 (33.7)	70 (23.2)	8 (8.8)	<0.0001
High	493 (74.5)	179 (66.3)	232 (76.8)	82 (91.2)	
Pathological T stage					
≤pT2	338 (51.1)	164 (60.7)	145 (48.0)	29 (32.2)	<0.0001
≥pT3	324 (48.9)	106 (39.3)	157 (52.0)	61 (67.8)	
Lymph node status					
pN0/X	598 (93.0)	247 (91.5)	271 (89.7)	80 (88.9)	0.689
pN+	64 (9.7)	23 (8.5)	31 (10.3)	10 (11.1)	
LVI					
No	562 (84.9)	244 (90.4)	247 (81.8)	71 (78.9)	0.004
Yes	100 (15.1)	26 (9.6)	55 (18.2)	19 (11.1)	
Tumor size (cm)					
<3	212 (32.0)	85 (31.5)	104 (34.4)	23 (25.6)	0.276
≥3	450 (68.0)	185 (68.5)	198 (65.6)	67 (74.4)	
PSM					
No	608 (91.8)	253 (93.7)	278 (92.1)	77 (85.6)	0.049
Yes	54 (8.2)	17 (6.3)	24 (7.9)	13 (14.4)	
Tumor architecture					
Papillary	206 (31.1)	106 (39.3)	82 (27.2)	18 (20.0)	<0.0001
Sessile	456 (68.9)	164 (60.7)	220 (72.8)	72 (80.0)	
CVH					
No	513 (77.5)	221 (81.9)	233 (77.2)	59 (65.6)	0.006
Yes	149 (22.5)	49 (18.1)	69 (22.8)	31 (34.4)	
Postoperative treatment					
Adjuvant chemotherapy					
No	383 (57.9)	151 (22.8)	168 (25.4)	64 (9.7)	0.023
Yes	279 (42.1)	119 (18.0)	134 (20.2)	26 (3.9)	
Adjuvant radiotherapy					
No	618 (93.4)	253 (93.7)	279 (92.4)	86 (95.6)	0.545
Yes	44 (6.6)	17 (6.3)	23 (7.6)	4 (4.4)	
Serum albumin, g/L	39.8 ± 5.1	42.2 ± 3.3	39.9 ± 3.9	32.1 ± 5.4	<0.0001
Total lymphocytes, /mm^3^	1485 ± 578	1898 ± 472	1305 ± 448	849 ± 309	<0.0001
Total cholesterol, mg/dL	169.5 ± 37.8	193.2 ± 32.6	158.4 ± 30.0	135.8 ± 33.1	<0.0001

BMI, body mass index; CVH, concomitant variant histology; LVI, lymphovascular invasion; PSM, positive surgical margins; RNU, radical nephroureterectomy.

### 3.2 Associations of CONUT score with pathological features

We have performed the multivariate logistic regression analysis to explore the associations of CONUT score with adverse pathological outcomes. After adjusting preoperative confounders including age, gender, BMI, hydronephrosis, tumor side, tumor location, and multifocality, our results demonstrated that high CONUT score (mild vs normal or moderate/severe vs normal) was significantly associated with high‐grade disease (moderate/severe vs normal: odds ratios [OR] 5.5, 95% confidence interval [CI] 2.53‐11.99, *P* < 0.0001), high pT stage (OR 3.64, 95% CI 2.16‐6.13, *P* < 0.0001), LVI (OR 2.61, 95% CI 1.35‐5.04, *P* = 0.004), sessile carcinoma (OR 2.56, 95% CI 1.43‐4.59, *P* = 0.002), and PSM (OR 2.98, 95% CI 1.34‐6.59, *P* = 0.007), except for lymph node involvement (OR 1.39, 95% CI 0.62‐3.08, *P* = 0.422; Table 3).

**Table 3:** Odds ratios for CONUT score for pathological outcomes when adjusting preoperative factors

Adverse pathological outcomes	Adjusted OR^a^	95% CI	*P* value
High‐grade disease	*P* < 0.0001		
Light vs normal	1.78	1.22‐2.60	0.003
Moderate/severe vs normal	5.50	2.53‐11.99	<0.0001
High pT stage (≥pT3)	*P* < 0.0001		
Light vs normal	1.81	1.28‐2.54	0.001
Moderate/severe vs normal	3.64	2.16‐6.13	<0.0001
Lymph node involvement	*P* = 0.587		
Light vs normal	1.31	0.74‐2.34	0.357
Moderate/severe vs normal	1.39	0.62‐3.08	0.422
LVI	*P* = 0.004		
Light vs normal	2.09	1.26‐3.47	0.004
Moderate/severe vs normal	2.61	1.35‐5.04	0.004
Sessile carcinoma	*P* < 0.0001		
Light vs normal	1.86	1.30‐2.68	0.001
Moderate/severe vs normal	2.56	1.43‐4.59	0.002
CVH	*P* = 0.007		
Light vs normal	1.35	0.89‐2.05	0.160
Moderate/severe vs normal	2.41	1.40‐4.16	0.002
PSM	0.025		
Light vs normal	1.36	0.7‐2.66	0.361
Moderate/severe vs normal	2.98	1.34‐6.59	0.007

CI, confidence interval; CVH, concomitant variant histology; LVI, lymphovascular invasion; OR, odds ratios; PSM, positive surgical margins.

a Adjusting for age, gender, body mass index, hydronephrosis, tumor side, tumor location and multifocality.

### 3.3 Associations of CONUT score with survival outcomes

**Figure 1:**
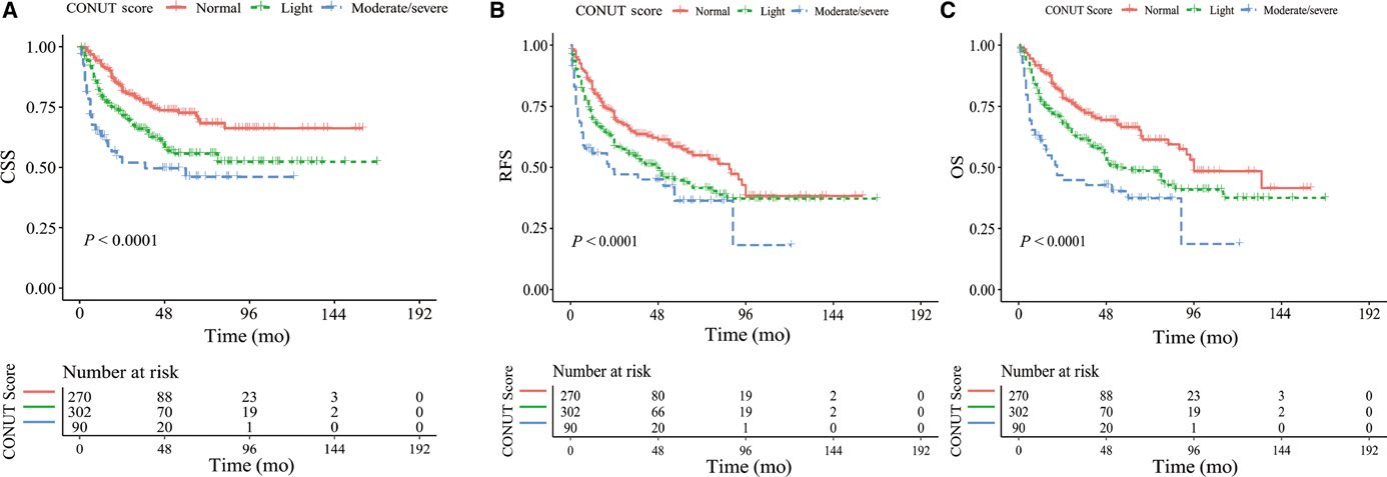
Kaplan‐Meier curves for survival in all UTUC patients according to the CONUT score. (A) cancer‐specific survival, (B) disease recurrence free survival and (C) overall survival

With the median follow‐up of 42 months (IQR 19‐72 months), 276 patients (41.7%) experienced disease recurrence, 239 (36.1%) died and 190 (28.7%) died of UTUC. The 5‐year CSS rates were 72.6%, 55.7%, and 46.1%, 5‐year. RFS rates were 58.5%, 44.8%, and 36.3%, and 5‐year. OS rate were 66.5%, 49.7%, and 37.3% in the normal, light and moderate/severe group, respectively. Kaplan‐Meier curves revealed that patients with higher CONUT score had significant worse survival compared with those who had normal CONUT score with respect to CSS, RFS, and OS (Log‐rank test, each *P* < 0.0001, Figure 1A‐C).

Univariate and multivariate Cox regression analysis (backward stepwise method) were performed in our study (Table S1 and Table 4). Univariate analysis demonstrated that tumor grade, tumor stage, lymph node status, LVI, tumor size, PSM, tumor architecture, CVH, and CONUT score were significant predictors for CSS, RFS, and OS (each *P* < 0.01). In addition, hydronephrosis was a significant predictor for RFS, but not for CSS and OS. Surgical approach was a prognostic factor for CSS and OS while not for RFS. Factors with the *P* value <0.05 were included in the multivariate Cox regression models by adopting backward stepwise method. These seven factors were included in the final models including tumor grade, pT stage, lymph node status, tumor size, tumor architecture, CVH, and CONUT score. The results showed that CONUT score was an independent predictor for CSS (Light vs normal: HR 1.69, 95% CI 1.21‐2.34; moderate/severe vs normal: HR 2.39, 95% CI 1.55‐3.68), RFS (Light vs normal: HR 1.43, 95% CI 1.10‐1.86; moderate/severe vs normal: HR 1.80, 95% CI 1.24‐1.60) and OS (Light vs normal: HR 1.58, 95% CI 1.18‐2.11; moderate/severe vs normal: HR 2.26, 95% CI 1.53‐3.34).

**Table 4:** Multivariable Cox regression models predicting survival outcomes in patients with upper tract urothelial carcinoma

Variables	Cancer‐specific survival				Recurrence‐free survival				Overall survival			
	HR (95% CI)	*P*	HR (95% CI)	*P*	HR (95% CI)	*P*	HR (95% CI)	*P*	HR (95% CI)	*P*	HR (95% CI)	*P*
Tumor grade (high vs low)	1.88 (1.15‐3.07)	0.012	1.72 (1.05‐2.83)	0.033	1.31 (0.93‐1.86)	0.123	1.25 (0.88‐1.77)	0.211	1.63 (1.09‐2.45)	0.018	1.50 (1.00‐2.26)	0.052
pT stage (≥pT3 vs ≤pT2)	2.19 (1.51‐3.17)	<0.0001	2.15 (1.48‐3.12)	<0.0001	2.00 (1.50‐2.68)	<0.0001	1.98 (1.48‐2.66)	<0.0001	2.10 (1.52‐2.89)	<0.0001	2.08 (1.50‐2.88)	<0.0001
Lymph node status (pN+ vs pN0/x)	2.22 (1.56‐3.17)	<0.0001	2.32 (1.62‐3.33)	<0.0001	2.30 (1.67‐3.16)	<0.0001	2.40 (1.74‐3.31)	<0.0001	2.08 (1.49‐2.90)	<0.0001	2.17 (1.54‐3.04)	<0.0001
Tumor size (≥3 cm vs <3 cm)	1.59 (1.13‐2.26)	0.009	1.74 (1.22‐2.47)	0.002	1.50 (1.13‐1.98)	0.005	1.58 (1.19‐2.10)	0.001	1.59 (1.17‐2.16)	0.003	1.71 (1.26‐2.33)	0.001
Architecture (Sessile vs Papillary)	1.88 (1.19‐2.96)	0.006	1.80 (1.14‐2.84)	0.012	1.50 (1.08‐2.10)	0.017	1.45 (1.04‐2.03)	0.030	1.60 (1.10‐2.32)	0.014	1.53 (1.05‐2.23)	0.001
CVH (yes vs no)	1.48 (1.08‐2.01)	0.014	1.36 (0.99‐1.87)	0.055	1.30 (1.00‐1.70)	0.054	1.24 (0.95‐1.63)	0.120	1.39 (1.05‐1.85)	0.021	1.30 (0.98‐1.73)	0.074
CONUT score				<0.0001				0.003				<0.0001
Light vs Normal			1.69 (1.21‐2.34)	0.002			1.43 (1.10‐1.86)	0.008			1.58 (1.18‐2.11)	0.002
Moderate/severe vs Normal			2.39 (1.55‐3.68)	<0.0001			1.80 (1.24‐2.60)	0.002			2.26 (1.53‐3.34)	<0.0001
Predictive accuracy	75.5%		77.2%		71.5%		72.3%		74.5%		75.6%	

CONUT, controlling nutritional status; CVH, concomitant variant histology; HR, hazard ratio.

The predictive accuracies of the multivariate models for survival outcomes considering whether CONUT score was supplemented were also assessed. In the base model, which included traditional variables of tumor grade, pT stage, lymph node status, tumor size, tumor architecture, and CVH, the predictive accuracy was 75.5%, 71.5%, and 74.5% for CSS, RFS, and OS, respectively. When the CONUT score was added, the predictive accuracy of the final model elevated to 77.2%, 72.3%, and 75.6% for CSS, RFS, and OS, respectively (Table 4).

### 3.4 Subgroup analyses

**Figure 2:**
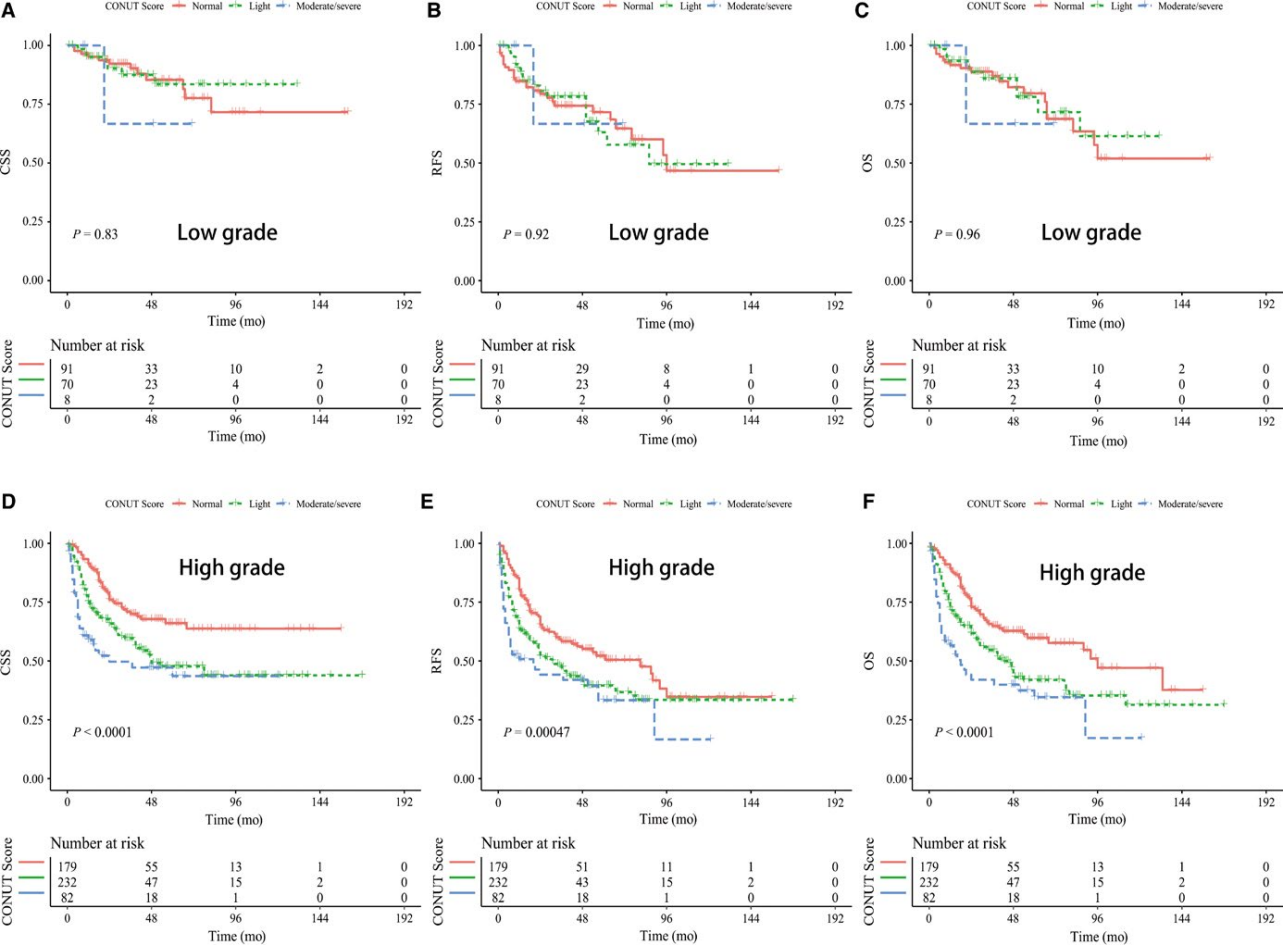
Kaplan‐Meier curves for cancer‐specific survival, disease recurrence‐free survival and overall survival stratified by CONUT score in UTUC patients with low‐grade carcinoma (A‐C) and high‐grade carcinoma (D‐F)

**Figure 3:**
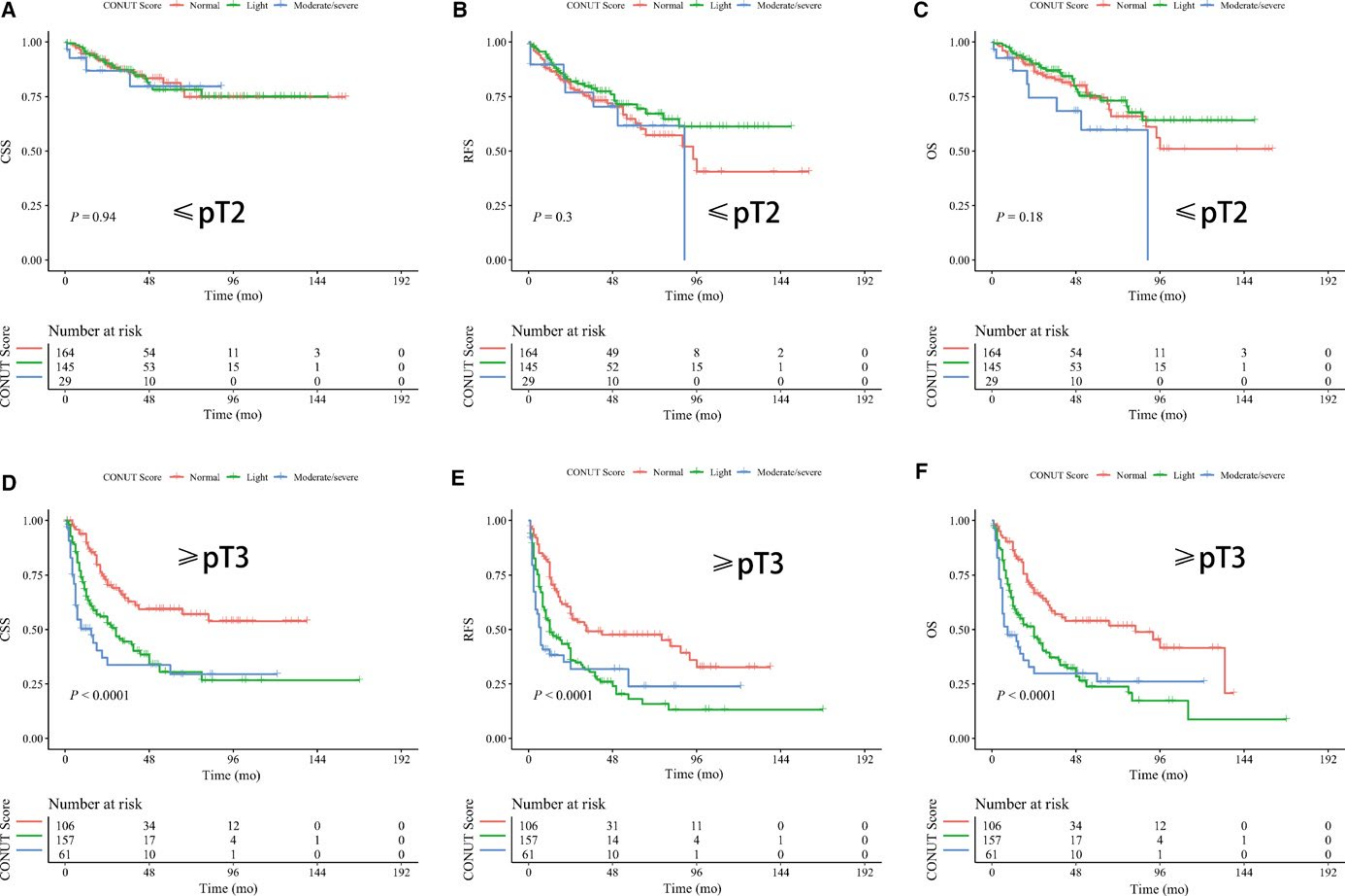
Kaplan‐Meier curves for cancer‐specific survival, disease recurrence‐free survival and overall survival stratified by CONUT score in UTUC patients with early stage (A‐C) and advanced stage (D‐F)

As for tumor grade and stage are the two most important factors in the prognosis of UTUC, we therefore further examined the prognostic role of CONUT score in UTUC patients stratified by tumor grade and pathological T stage. K‐M curves showed that patients with high CONUT score have significant lower CSS, RFS, and OS in those with high‐grade UTUC and advanced pT stage (log‐rank test, all *P* < 0.05, Figure 2D‐F and 3D‐F) while no significant differences were observed between groups in those with low‐grade UTUC and early stage (log‐rank test, all *P* > 0.05, Figures 2A‐C and 3A‐C). We also sought to explore whether the independent value of CONUT score would exhibit when considering tumor grade and stage. Table 5 showed the subgroup analyses of associations of CONUT score with survival outcomes. Our results revealed that high CONUT score can independently predict worse CSS, RFS and OS in high‐grade UTUC (HR: 2.65, 2.21, and 2.50, respectively) and in advanced UTUC (HR: 3.28, 2.40, and 2.79, respectively), while not in low‐grade UTUC and early stage UTUC (all *P* > 0.05; Table 5).

**Table 5:** Subgroup analyses of associations of CONUT score with survival outcomes when stratified by tumor grade and stage

Subgroups	CSS		RFS		OS	
	Unadjusted	Adjusted^a^	Unadjusted	Adjusted^a^	Unadjusted	Adjusted^a^
With high‐grade carcinoma; CONUT score						
Normal (0‐1)	Ref.	Ref.	Ref.	Ref.	Ref.	Ref.
Light (2‐4)	1.78 (1.25‐2.53)	1.92 (1.34‐2.75)	1.48 (1.10‐1.98)	1.60 (1.19‐2.15)	1.64 (1.20‐2.25)	1.83 (1.33‐2.53)
Moderate/severe (5‐12)	2.69 (1.73‐4.19)	2.65 (1.67‐4.19)	2.04 (1.40‐2.99)	2.21 (1.49‐3.26)	2.54 (1.71‐3.79)	2.50 (1.65‐3.77)
With low‐grade carcinoma; CONUT score						
Normal (0‐1)	Ref.	Ref.	Ref.	Ref.	Ref.	Ref.
Light (2‐4)	0.89 (0.35‐2.27)	1.33 (0.45‐3.88)	0.97 (0.53‐1.76)	1.08 (0.56‐2.08)	0.92 (0.44‐1.93)	1.27 (0.55‐2.91)
Moderate/severe (5‐12)	1.69 (0.22‐13.1)	1.28 (0.09‐17.3)	0.67 (0.09‐4.95)	0.57 (0.07‐4.62)	1.13 (0.15‐8.52)	1.27 (0.14‐11.3)
With advanced stage(≥pT3); CONUT score						
Normal (0‐1)	Ref.	Ref.	Ref.	Ref.	Ref.	Ref.
Light (2‐4)	2.11 (1.41‐3.15)	2.30 (1.53‐3.45)	1.92 (1.37‐2.69)	1.95 (1.39‐2.75)	2.06 (1.44‐2.95)	2.21 (1.53‐3.20)
Moderate/severe (5‐12)	3.15 (1.94‐5.10)	3.28 (1.99‐5.40)	2.25 (1.46‐3.46)	2.40 (1.53‐3.75)	2.74 (1.75‐4.28)	2.79 (1.74‐4.45)
With early stage (≤pT2); CONUT score						
Normal (0‐1)	Ref.	Ref.	Ref.	Ref.	Ref.	Ref.
Light (2‐4)	1.04 (0.58‐1.86)	0.80 (0.42‐1.55)	0.74 (0.47‐1.15)	0.72 (0.46‐1.13)	0.83 (0.50‐1.37)	0.74 (0.44‐1.24)
Moderate/severe (5‐12)	1.22 (0.42‐3.52)	1.44 (0.45‐4.67)	1.17 (0.55‐2.49)	1.37 (0.63‐2.95)	1.73 (0.80‐3.75)	1.94 (0.88‐4.27)

CONUT, controlling nutritional status; CSS, cancer‐specific survival; OS, overall survival; RFS, recurrence‐free survival.

Data presented as hazard ratio (95% CI) unless otherwise noted.

a Stepwise selection procedure (*P* < 0.2 for entry; *P* < 0.15 to remain) for Cox regression models adjusting for potential confounders.

## 4 DISCUSSION

In this retrospective study, we enrolled 662 patients of Chinese origin with UTUC after RNU and we demonstrated that CONUT score was an independent predictor for worse oncological outcomes. High CONUT score had significant lower CSS, RFS, and OS rates compared with normal CONUT score, and this phenomenon was more pronounced in patients with high grade and advanced stage of UTUC. Moreover, the addition of CONUT score included in the multivariate model would improve its predictive accuracy. To the best of our knowledge, this study is the largest to address the relationships between CONUT score and oncological outcomes in UTUC.

Probing the prognostic value of CONUT score in cancer patients is never new. Actually, it has been proved that CONUT score was an independent factor for decreased survival in many cancer types,^8^, ^9^ and a recent meta‐analysis which included four publications of 674 patients also found that high CONUT score independently predicted worse OS in patients with solid tumors.^14^ In the context of the low incidence of UTUC, researches in this field are still limited. Our results were in line with these studies which support the CONUT score as an independent predictor for survival outcomes. To date, only one study which is conducted by Ishihara et al^7^ was available in assessing the prognostic value in UTUC sets. Similarly, we both demonstrated that CONUT score is an independent factor for both CSS and OS. Nevertheless, owing to the limited number of cases (only 107 cases) included in their study, they failed to reveal the independent value of CONUT score for RFS (HR 2.26, 95% CI 0.97‐4.94), which exhibited statistical difference in our study not only in the entire cohort (moderate/severe vs normal: HR 1.80, 95% CI 1.24‐1.60), but in the high‐grade UTUC (HR 2.21, 95% CI: 1.49‐3.26) and advanced stage (HR 2.40, 95% CI: 1.53‐3.75). Furthermore, our detailed subgroup analysis and the calculation of c‐index enable us to get a better understanding of the role of CONUT score in UTUC.

CONUT score is calculated from serum albumin, total lymphocyte counts, and total cholesterol concentrations. Albumin is a major component of serum total proteins and it is a reflection of both nutrition and inflammation status.^15^, ^16^ Hypoalbuminemia (low serum albumin level) could decrease individual’s immunity and lead to poor oncological outcomes.^17^ Researches also certified the negative prognostic role of hypoalbuminemia in UTUC patients,^18^, ^19^ and our previous study also showed the independent prognostic of albumin to globulin ratio in UTUC.^5^ In addition, lymphocytes function in host immunity and are considered to have antitumor ability via affecting tumor cell growth, migration, apoptosis, and inducing cytotoxicity.^21^ Previous study indicated that the infiltration of lymphocyte (CD4^+^/CD8^+^ T‐lymphocytes) might affect patients’ response for neoadjuvant chemotherapy in urothelial carcinoma of the bladder,^22^ and our previous work also revealed that low preoperative neutrophil to lymphocyte ratio was independently associated with worse survival outcomes.^4^ In addition, low serum cholesterol levels was also associated with poor survival in colorectal cancer^23^ and renal cell carcinoma,^24^, ^25^ but the specific mechanism remained to be elucidated. In all, CONUT, a combination of these three indictors above, is more powerful and can be applicated in current risk stratifications of UTUC.

Malnutrition is a common phenomenon which can be found in cancer patients and previous evidence support that malnutrition was associated with worse outcomes.^26^ In the study conducted by Naito et al^27^ they defined CONUT score 5‐12 as malnutrition status. Our results showed that patients with CONUT score 5‐12 had significant worse survival rate that those with normal CONUT score, especially in those with high grade and advanced stage, providing another strong evidence regarding the negative role of CONUT in UTUC. Most importantly, in comparison of the traditional pathological factors, CONUT score can be accessed easily and cost‐effectively from which way it allowed the possibility of preoperative intervention to achieve the better tumor control effects.

Limitations should be noticed as well. First, as with all retrospective studies, the selection bias could not be avoided. Second, other potential inflammation indicators (eg, CRP) were not routinely measured before their hospitalization and their medication information was incomplete, which might affect our results. Third, although we have excluded patients with autoimmune status or hepatitis, other potential disease which was not detected preoperatively might affect the level of albumin, lymphocyte, and total cholesterol. Fourth, our results also showed that the surgical procedure and adjuvant chemotherapy would not affect patients’ survival in UTUC, they should be further validated in large‐scale multicenter prospective clinical trials. Last, although our data showed that the addition of CONUT score would improve the models’ predictive accuracy, the model validity (internal and external validation) is still warranted so as to assist clinical practice.

## 5 CONCLUSIONS

In this study we included a large UTUC group of Chinese origin to validate the prognostic value of CONUT score in UTUC after RNU. We finally demonstrated CONUT score independently predicted worse survival in UTUC patients, especially in patients with high‐grade disease and advanced stage. The addition of CONUT score would improve the predictive accuracy of the multivariate models for survival outcomes. We therefore suggest adding the CONUT score to the traditional predictive model and to the risk stratifications of patients with UTUC.

## ETHICAL APPROVAL

All procedures performed in studies involving human participants were in accordance with the ethical standards of the institutional and/or national research committee and with the 1964 Helsinki declaration and its later amendments or comparable ethical standards. For this type of study, formal consent is not required.

## CONFLICT OF INTERESTS

All authors declare no conflict of interests.
