# Supplementary material for: Validation of the preoperative controlling nutritional status score as an independent predictor in a large Chinese cohort of patients with upper tract urothelial carcinoma
Source: Cancer Med. 2018 Nov 28;7(12):6112–23. doi: 10.1002/cam4.1902 (PMC6308095; doi:10.1002/cam4.1902)
Supplement: Supplementary file 1 [file CAM4-7-6112-s001.doc]

Table S1. Univariable Cox regression analyses predicting survival outcomes in patients with upper tract urothelial carcinoma.

| Variables | Cancer-specific survival | | Recurrence-free survival | | Overall survival | |
| --- | --- | --- | --- | --- | --- | --- |
|  | HR (95%CI) | P | HR (95%CI) | P | HR (95%CI) | P |
| Age (≥ 65 years vs < 65 years) | 0.75 (0.56-1.00) | 0.052 | 0.79 (0.62-1.00) | 0.051 | 0.87 (0.67-1.13) | 0.293 |
| Gender (male vs female) | 0.86 (0.64-1.14) | 0.285 | 0.90 (0.71-1.15) | 0.406 | 0.92 (0.71-1.19) | 0.519 |
| BMI (≥ 25 vs < 25) | 0.89 (0.65-1.23) | 0.481 | 0.99 (0.76-1.28) | 0.937 | 0.95 (0.72-1.26) | 0.739 |
| Tumor side (right vs left) | 1.08 (0.82-1.44) | 0.584 | 1.06 (0.84-1.35) | 0.615 | 1.06 (0.82-1.37) | 0.643 |
| Hydronephrosis (yes vs no) | 1.21 (0.89-1.63) | 0.225 | 1.33 (1.03-1.72) | 0.027 | 1.28 (0.97-1.67) | 0.079 |
| Tumor location |  | 0.679 |  | 0.533 |  | 0.696 |
| Ureteric vs Pelvicalyceal | 0.96 (0.69-1.34) | 0.824 | 0.89 (0.67-1.18) | 0.418 | 0.90 (0.67-1.22) | 0.490 |
| Both vs Pelvicalyceal | 1.16 (0.79-1.69) | 0.456 | 1.09 (0.79-1.50) | 0.608 | 1.05 (0.74-1.49) | 0.777 |
| Multifocality (yes vs no) | 1.04 (0.72-1.52) | 0.823 | 0.95 (0.69-1.31) | 0.755 | 0.95 (0.67-1.34) | 0.770 |
| Surgical approach (Laparoscopic vs Open) | 0.67 (0.48-0.93) | 0.018 | 0.86 (0.66-1.12) | 0.270 | 0.72 (0.53-0.97) | 0.029 |
| Tumor grade (high vs low) | 3.53 (2.22-5.61) | < 0.0001 | 2.16 (1.57-2.98) | < 0.0001 | 2.80 (1.92-4.09) | < 0.0001 |
| pT stage ((≥ pT3 vs ≤ pT2) | 4.19 (3.03-5.81) | < 0.0001 | 3.18 (2.47-4.10) | < 0.0001 | 3.62 (2.73-4.79) | < 0.0001 |
| Lymph node status (pN+ vs pN0/x) | 4.09 (2.90-5.77) | < 0.0001 | 3.73 (2.75-5.07) | < 0.0001 | 3.63 (2.62-5.01) | < 0.0001 |
| LVI (yes vs no) | 2.64 (1.91-3.65) | < 0.0001 | 2.12 (1.59-2.81) | < 0.0001 | 2.41 (1.79-3.23) | < 0.0001 |
| Tumor size (≥ 3 cm vs < 3 cm) | 2.06 (1.47-2.89) | < 0.0001 | 1.85 (1.41-2.43) | < 0.0001 | 2.00 (1.48-2.69) | < 0.0001 |
| PSM (yes vs no) | 2.23 (1.46-3.39) | < 0.0001 | 1.77 (1.21-2.59) | 0.003 | 2.02 (1.37-2.97) | < 0.0001 |
| Tumor architecture (Sessile vs Papillary) | 3.78 (2.48-5.68) | < 0.0001 | 2.51 (1.86-3.38) | < 0.0001 | 2.92 (2.09-4.09) | < 0.0001 |
| CVH (yes vs no) | 2.38 (1.77-3.22) | < 0.0001 | 1.98 (1.53-2.57) | < 0.0001 | 2.14 (1.64-2.81) | < 0.0001 |
| Adjuvant Chemotherapy (yes vs no) | 1.02 (0.89-1.18) | 0.745 | 0.95 (0.84-1.06) | 0.355 | 1.06 (0.94-1.21) | 0.356 |
| Adjuvant radiotherapy (yes vs no) | 1.50 (0.71-3.17) | 0.288 | 1.05 (0.97-1.14) | 0.228 | 1.56 (0.77-3.16) | 0.217 |
| **CONUT score** |  | < 0.0001 |  | < 0.0001 |  | < 0.0001 |
| Light vs Normal | 1.75 (1.27-2.42) | 0.001 | 1.41 (1.09-1.83) | 0.010 | 1.59 (1.20-2.12) | 0.001 |
| Moderate/severe vs Normal | 3.05 (2.01-4.65) | < 0.0001 | 2.12 (1.48-3.04) | < 0.0001 | 2.79 (1.91-4.06) | < 0.0001 |

BMI, body mass index; LVI, lymphovascular invasion; CVH, concomitant variant histology; PSM, positive surgical margins; CONUT, controlling nutritional status; HR, hazard ratio.
